# Awareness of Environmental Change, Climate Variability, and Their Role in Prevalence of Mosquitoes among Urban Dwellers in Southern Ghana

**DOI:** 10.1155/2018/5342624

**Published:** 2018-05-07

**Authors:** Precious Agbeko Dzorgbe Mattah, Godfred Futagbi, Memuna Mawusi Mattah

**Affiliations:** ^1^Institute of Environment and Sanitation Studies (IESS), University of Ghana, Legon, Ghana; ^2^Centre for Coastal Management (CCM), University of Cape Coast, Cape Coast, Ghana; ^3^Department of Animal Biology and Conservation Science, University of Ghana, Legon, Ghana; ^4^Department of Environment and Development Studies, Central University, Accra, Ghana

## Abstract

This study evaluates the extent to which urban residents of Accra and Sekondi-Takoradi (all of southern Ghana) were aware of environment and climate-related variability. A total of 150 questionnaires were given to adults of 40 years and above who lived in the cities for at least 35 years. SPSS version 16 was used to process the data. Results show that all respondents in Accra Metropolitan Area (AMA) and 96% of them in Sekondi-Takoradi Metropolitan Area (STMA) noted the deteriorating vegetation cover over the cities. Similarly, 93% and 83% of them in AMA and STMA, respectively, observed that land use pattern of the two cities has changed considerably. There was positive and statistically significant correlation between respondents' educational attainment and their awareness of changes in land use/land cover (*r* = 0.18, *n* = 140, *p* = 0.03). Also, 71% in AMA and 89% in STMA stated that temperature of the cities had been increasing over the years. In all, 82.9% of the respondents mentioned that they had problem with the increasing population of mosquitoes. Respondents demonstrated ample knowledge of environment and climate variability and should be engaged in preventing further environmental degradation. The top-down approach to environmental issues has failed; the bottom-up approach to environmental maintenance is needed.

## 1. Introduction

Rapid urbanization and climate variability are two important phenomena affecting human development and well-being in the 21st century [1]. Whether separately or combined, these phenomena pose devastating threats to all societies especially those in less developed countries [1, 2]. Rapid urbanization for example, has numerous environmental effects which are felt at different but interconnected scales. The scales range from those that affect the home to those that operate at regional or continental levels [2–4]. At the regional or continental level, effects such as inducement of climate change [5, 6], deterioration of water quality [7, 8], increase in air pollution [9], and the destruction of the natural habitat and ecosystems [10] have been noted. Among the effects associated with the home are the numerous health-related issues mostly experienced by poor urban households. Poor living conditions in rapidly urbanized environments encourage the prevalence and proliferation of pathogens and vectors that cause many debilitating and endemic diseases [2, 3]. Climate variability, on the other hand, may be responsible for droughts, erratic rainfalls, floods, storm surges, temperature rise, and sea level rise that often results in the destruction of human habitats especially urban areas [1]. In fact, cities of Africa are reportedly among the most vulnerable in terms of exposure, sensitivity, and capacity to respond to climate variability-related disasters worldwide [11]. This is because of their limited capacity to recover from such occurrences [1].

Studies have noticed positive correlation between urban growth and environmental changes in Africa [12–14]. According to Linard et al. [15], the list of changes associated with urban growth is endless and include land use and land cover changes, biogeochemical changes, and changes in hydrological cycles among others. However, most of these studies were done in the form of land use and land cover change using remotely sensed imageries and scarcely from the perspective of local residents of the study areas. Though nascent in the climate change related sciences, the importance of local community residents' (including urban dwellers) knowledge in environment-based fields such as agroforestry and biodiversity, among others, cannot be underestimated [16]. Importantly, their observations and assessments provide useful in situ information that may enhance local verification and validation of scientific models and satellite imageries. Local residents' knowledge is considered as a place-based tool for ground truth of climate models in order to narrow the geographic sensitivity of models [17–19]. According to Stigter et al. [20] and Fernandez-Llamazares et al. [21], local residents who have a long history of interaction with their environments have good knowledge on weather and the climate variabilities of their areas. It therefore means that local residents need to reside in the area for a long period of time to have good knowledge of the place. They therefore observe and are most often privy to changes relating to their environment (physical, biological, and socioeconomic) and the climate changes occurring. From literature, it has been observed that changes in climate variables such as precipitation and temperature, just as used in scientific literature, have been used by local residents as indicators of climate change [19]. Descriptions such as “rising temperatures” [22], “dry season temperature increase” [23], and “sudden fluctuations in temperature” [24] were reportedly used by local residents in certain jurisdictions to describe climate variability. These terms, which are derived mainly from opinions of local residents, are considered as local observations in climate change [19]. Several years or decades of interactions with their immediate environment provide them with the ability to describe the changes occurring. Increasingly, local residents have been recognized as stakeholders whose knowledge can be tapped to enhance the understanding of climate variability and its impact [25, 26]. This paper therefore dwelt strongly on the opinions of residents of two coastal urban areas of Ghana to determine if they (the residents) were aware of changes occurring in their environment.

Awareness of environmental changes induced by urbanization and climate variability vary greatly from one society to the other worldwide [27]. An understanding of public perception about urban-induced environment and climate variability may provide strategic directions for government policies and informed choices for communities. This paper examines the extent of awareness among residents of Accra (AMA) and Sekondi-Takoradi (STMA) Metropolitan Areas of Ghana regarding land use, land cover change, and climate variability and how these phenomena could be linked to the prevalence of certain disease vectors such as mosquitoes in the cities. The study also sought to know whether the respondents could identify specific weather and environmental events (in specific years) that point to an ongoing environmental change and climatic variability.

## 2. Methodology

### 2.1. Study Areas

**Figure 1:**
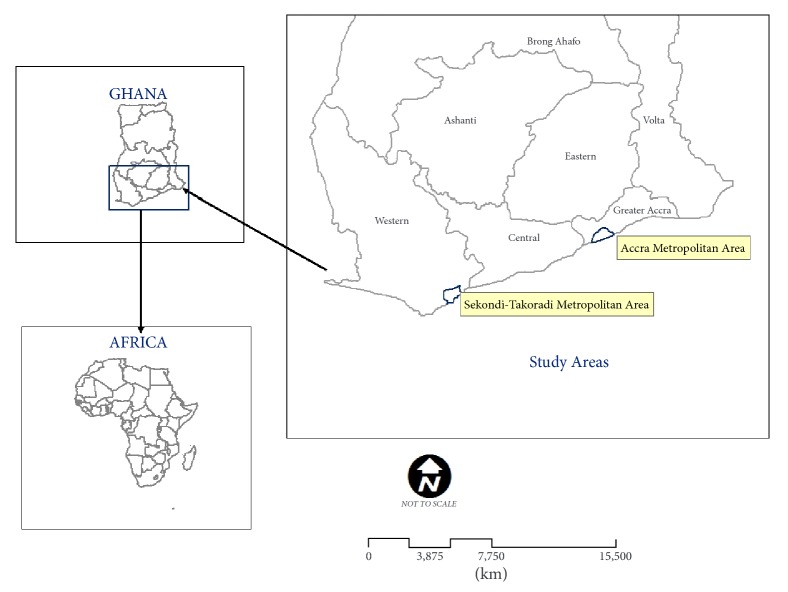
Location of Accra and Sekondi-Takoradi Metropolitan Areas.

The study was conducted in Accra Metropolitan Area (AMA) and Sekondi-Takoradi Metropolitan Area (STMA) of Ghana in 2013/2014 as part of a larger study that was done to examine the impact of environment and climate variability on certain mosquito species. The two areas are the largest metropolitan areas along the coast of Ghana and for that matter presented suitable settings for a study on the interactions between environment, climate, disease vectors, and human populations in the urban milieu.  Figure 1 shows the location of the two cities.

Accra Metropolitan Area (AMA) currently covers an approximate land surface area of 120 km^2^. It is located on latitude 5°32′′N and longitude 0°13′′W. With a population of over 1,848,614 million people, the population density is about 895.5 persons per kilometer with a growth rate of 4.4% as at 2010 [28]. Accra lies within the coastal savanna agroecological zone which has two rainfall seasons. The major rainy season is between April and June and the minor season is from September to October. The average annual rainfall is 730 mm and rainfall in the capital is characterized by intensive but short storms which often lead to flooding especially in areas where drainage systems have been obstructed [29]. The average annual temperature of AMA is 26.8°C. While March is the hottest month with 28°C, the coldest month is August with 24.7°C on the average. The metropolis has three main vegetation types: (i) shrub land which is mainly found in the western outskirts (Weija areas) and the northern part of the city towards Aburi and comprises dense clusters of small trees and shrubs not more than 5 m high, (ii) the grassland, which is found around the edges of the shrubs, characteristically short, not taller than one metre and typical of the type found underneath forest covers, and (iii) coastal lands made up of mangroves and grasses associated with brackish water environment [29]. The general soil types in Accra are the drift materials, alluvial and marine mottled clay, residual clays and gravels, and lateritic sandy clay soil [29].

Sekondi-Takoradi Metropolitan Area (STMA) is located on latitude 4° 55′′N and longitude 1°46′′W and about 280 km west of Accra, the capital of Ghana. It is relatively small in size (about 49.78 sq. km) compared to AMA, but the most developed (in terms of infrastructure) among the 17 districts of the Western Region. The metropolis is home to 23.5% of the entire population (2.3 million people) of the Western region [28]. According to the Ghana Statistical Service [28], STMA is the third largest metropolis (in terms of population) in Ghana having a total of 559,548 people after Kumasi Metropolitan Area (pop. 2,035,064) and Accra Metropolitan Area (pop. 1,848,614). STMA has a mean annual rainfall of 2,350 mm with an average daily temperature of 22°C. Rainfall is of double maxima with the major rainy season from April to June and the minor season between September and October. The vegetation is of the deciduous forest type characterized by tall trees, interspersed with grass cover, shrubs, and soft woody species mainly along the coast [30].

### 2.2. Data Collection

A semistructured questionnaire was developed and pretested on 20 randomly selected participants in the Cape Coast Metropolitan Area of Ghana. The pilot group was asked to comment on how comprehensible the individual questions were. The pretest led to some modification of the questionnaire. The questionnaire was divided into six sections: background, vegetation, land use, weather, rivers/streams/drainage, and presence of mosquitoes. The first section provided information on the background of the respondents including age, sex, marital status, highest level of education, employment, occupation, and the number of years that respondents had lived in the cities. The section on vegetation requested a description of the vegetation coverage over the city from the respondents. It sought to know whether the respondents noticed changes in the vegetation and what contributed to the changes if they noticed changes. Issues on land use pattern sought to find out the dominant land uses in the city in the past and the present and whether the respondents noticed changes that were occurring. Respondents having generally been living in the areas for more than three decades were quizzed on their observations on the weather/climate patterns of the cities regarding temperature/heat and rainfall as well as the perceived variations in these variables over the years. The state of rivers/streams and drainage patterns of the cities were brought into focus in order to ascertain whether there were changes over the years. The section on the presence of mosquitoes was to enable respondents to provide information on the prevalence of mosquitoes possibly as a factor of environmental changes occurring in the cities.

Sampling of respondents was fit into a larger sampling scheme as described in Mattah et al. [31], in which points, two (2) kilometers from each other, were overlaid on maps of the study areas using R software [32] and ArcGIS (ESRI, Redlands, California). These points served as the sampling frame out of which 20% were randomly selected using the random sample of cases in SPSS version 16 (SPSS Inc. Chicago, USA). Respondents were selected from suburbs/communities nearest to the presampled points. Because local residents must inhabit the area long enough to have good knowledge of the changes that might have occurred in their environment [20, 21], criteria for selection of respondents were that they should be males and females who attained a minimum of 40 years and must have continuously lived in the cities for not less than 35 years. A total of 150 questionnaires were divided equally between the two cities and given to those who met the sampling criteria.

### 2.3. Statistical Analysis

SPSS version 16 was used to capture, clean, and analyze the data collected. Descriptive statistics was mainly employed in the analysis of the data. Categorical variables were measured as percentages, while continuous variables were summarized using mean or median. Linear correlation was used to ascertain the association of respondents' educational attainment and their awareness of changes in land use/land cover. Z-test for proportions was used to compare proportions between the two cities. Sociodemographic variables and responses from study participants have been summarized and presented in frequency tables.

### 2.4. Ethical Approval and Consent

The study protocol was approved by the Ethical Review Board of the Noguchi Memorial Institute of Medical research (NMIMR) of the University of Ghana, Legon. Written informed consent was sought and obtained from the respondents after the written information was read out at the beginning of the interview.

## 3. Results

### 3.1. Characteristics of Respondents

Most of the 150 respondents were males (53%) with the proportion of male respondents in AMA and STMA being 27% and 26%, respectively (Table 1). Also, 33% and 40% were married and resident in STMA and AMA, respectively. Two percent (2%) apiece of the respondents from AMA and STMA were cohabiting, while 5.4% and 1.4% of them were divorced in AMA and STMA, respectively. In the AMA sample, 15.4% of the respondents had no education, while 16.8% and 16.1% attained primary and secondary education, respectively, and only 2% of them obtained tertiary education. A proportion of 12.8% respondents had no education, while those who had primary, secondary, and tertiary education were 15.4%, 17.4%, and 4%, respectively, in the STMA sample. The mean age of all respondents was 57.6 (±11.2) years. Put together, 20% of the respondents from the two cities were in the age group of 50–54, while a minimum proportion of 5.3% were within 40–44 age group, also from the two cities. Respondents from AMA had a mean age of 56.6 (±11.5) years, while those from STMA had a mean age of 58.3 (±10.9) years. Majority (24%) of the respondents had been living in AMA and STMA (combined) for the past 35–39 years, while 3.3% had lived in the cities for 65–69 years (Table 1). The mean duration of stay in the two cities combined was 46 years (median = 45, interquartile range (25th–75th) = 39–54). For AMA, the mean duration of stay in the city by the respondents was 44.6 years (median = 43, interquartile range (25th–75th) = 37–50) and that of STMA was 48.5 (median = 47.5, interquartile range (25th–75th) = 40–55).

### 3.2. Respondents' Views on Environmental Changes

Respondents, who had lived in the cities for at least 35 years, noticed various environmental changes occurring in the two cities. Environmental changes identified included a remarkable increase in built-up areas resulting in land use/land cover changes and destruction of streams/rivers. From the data, 93% and 83% of the respondents from AMA and STMA, respectively, observed changes in land use/land cover patterns in the cities. There was a weak positive but statistically significant correlation between respondents' educational attainment and their awareness of changes in land use/land cover (r = 0.18, n = 140, p = 0.03). Also, all respondents (100%) in AMA and 96% of them in STMA noticed changes in the vegetation cover of the cities (Table 2). For example, forest/conservation areas and agricultural/farmlands dwindled over the years according to the respondents, while residential areas and commercial land uses such as markets/shops and office buildings, among others, have increased within the same spate of time. Higher proportions of respondents, 77% from AMA and 86% from STMA, remembered parts of the metropolis which had been covered with vegetation but were cleared for other land uses. There was no statistical significant difference at p < 0.05 between the proportion of respondents in AMA who remembered areas which had earlier been covered with vegetation and that of STMA in a Z-test for independent proportions, Z-score = −0.765, p = 0.45, two tailed. A similar test indicated no significant association at p < 0.05 between gender and respondents' ability to remember areas that had earlier been covered with vegetation but were cleared for other land uses, Z-score = 0.43, p value = 0.67, two tailed. Respondents recalled that suburbs, such as Alajo in AMA and Kwesimintsim in STMA, were farming areas for some residents in the past especially in 1960s but they are now part of the urban sprawl. Table 2 further shows that 75% and 81% of the respondents from AMA and STMA, respectively, noticed changes in streams/rivers and other water bodies in the two metropolitan areas. According to the respondents, siltation and acute pollution were the two important phenomena affecting streams/rivers of the cities. In their views, poor waste management and lack of waste treatment measures by residents and municipal authorities have contributed to extreme pollution along water courses in the two metropolitan areas. From the respondents' perspective rapid urbanization with its concomitant issues of housing and infrastructural development was the main cause of degradation in the vegetative cover as well as land use and land cover changes in the cities.

Table 3 summarizes the observations of the respondents as far as environmental changes were concerned, on decadal basis. Starting from the decade of 1961 to 1970, respondents observed abundant grass/shrubs in most of AMA. The dominant tree species available was* Azadirachta indica* popularly called neem tree. There were few houses and numerous farming activities in the metropolis. Respondents from STMA observed vast forest cover, numerous coconut trees, intense farming activities, and few houses. The decade of 1971 to 1980 witnessed some increase in housing facilities and farming activities, yet abundant grasslands were available in AMA. However, for STMA in the same spate of time forest depletion started, housing facilities increased in the midst of faming activities. The rest of the decades (1981–1990; 1991–2000; and 2001–2010) saw rapid expansion of the cities as shown by the increase in housing facilities and especially the rise in uncoordinated buildings, depletion of the forests, reduction in farming activities, and the rise of slums or shanty towns.

### 3.3. Respondents' Views on Weather/Climate Variability

Table 4 summarizes the views of the respondents on environmental temperature and amount of rainfall. Most respondents, 71% in AMA and 89% in STMA observed that temperature of the cities has consistently been increasing over the years. Overall, 79.5% of the respondents perceived that temperature over the cities has been increasing. Twenty percent (20%) of the respondents from AMA and 7% from STMA thought that environmental temperature had fluctuated over the years, while a very small proportion (1.3% from AMA and 1.4% in STMA) felt that environmental temperature has consistently been reducing over the years. Comparing the decades from 1960 to 2010, the general perception of the respondents was that environmental temperature had been rising progressively from warm weather in the 1960s to an extremely warm one in the decade of 2000 to 2010. Warmer weather periods have even been experienced after 2010. From 1982, respondents began to feel the pinch of temperature increase. Specific years of extreme warm weather experienced by the respondents were in 1983 and 2013. Thirty-seven percent (37.1%) and 28.4% of the respondents from AMA mentioned 1983 and 2013, respectively. In STMA, 51.1% mentioned 1983, while 14.9% mentioned 2013 as years of extreme warmer temperatures.

Overall, about 70% of the respondents thought that the amount of rainfall in the cities had consistently been decreasing over the years (Table 4). In AMA, 22% of the respondents felt that the amount of rainfall had fluctuated, while about 17% of their counterparts in STMA also felt likewise. Though some found it difficult to mention a particular year in which they felt rainfall was extremely high, about 60% and 71% of the respondents in AMA and STMA, respectively, could recall certain specific years. When asked to mention the years, the list was long, starting from 1963 and included 2010 which is very recent. On the other hand, 40% and 47% of the respondents from AMA and STMA, respectively, recalled specific years with extremely low rainfall. Prominent among the list of years with low rainfall included 1983 mentioned by 61.5% of those who could recall specific years of drought from AMA and 50% of them from STMA.

### 3.4. Respondents' Views on Prevalence of Mosquitoes

Generally, respondents had problems with the abundance of mosquitoes. Overall, 82.9% of the respondents mentioned that they had problem with the increase in the population of mosquitoes. As high as 96.7% in AMA and 77.3% in STMA had problem with the proliferation of mosquitoes. The problems include mosquito frequent bites, noise with all the nuisance, and diseases especially malaria. Progressively higher proportions of the respondents claimed that mosquitoes were not widespread in the decade of 1961–1970 to being extremely abundant in the decade of 2001–2010 (Table 5). About 30.2% of the respondents thought that mosquitoes were not widespread within the decade of 1961–1970, while by 2001–2010, 34.9% of the respondents felt that mosquitoes were extremely pervasive. They associated mosquito breeding with the environment. In explaining their assertion, respondents indicated that general lack of care for the environment, coupled with human-induced changes such as the destruction of vegetation and construction activities, and climate variability were the cause of mosquito problems being experienced. According to them, indiscriminate dumping of refuse, poor sanitation, and choked drains in the various metropolitan areas were contributing to the proliferation of mosquitoes. Respondents associated prevalence of mosquitoes to the perennial floods in the cities especially Accra. They were of the view that floods leave pockets of stagnant water bodies which serve as breeding grounds of mosquitoes. Respondents felt that excessive heat during the dry season enable them to spend more time outside their rooms especially in the evenings and this expose them to mosquito bites.

## 4. Discussion

It is established that rapid urbanization enhances environmental change, and climate variability, phenomena that threaten the very existence of societies especially those in less developed countries [1]. Poor urban households are most vulnerable to the effects of environmental degradation [2]. Being aware of changes occurring in an environment provide the impetus to work at curtailing any negative effects that may emerge out of the change. This study sought to understand public perception on urban-induced environmental change and climate variability and whether participants could link identified weather and environmental events to the prevalence of diseases and disease vectors such as mosquitoes.

The results of this study provide insight into residents' consciousness of environmental change, weather/climate, and prevalence of mosquitoes in AMA and STMA of Ghana. Majority of the respondents had attained some form of education. Of the 150 respondents, almost one-third (32.2%) of the respondents had primary education, while another one-third (33.5%) had secondary education and 6% obtained tertiary level of education. The data has also shown a weak positive but significant correlation between level of education and perception of respondents regarding changes in land use/land cover in the cities. This is not surprising because educational attainment is seen as one important factor for environmental consciousness [33]. In fact majority of the respondents observed changes in vegetation cover, land use, and land cover as well as temperature and rainfall variability over the cities.

Rapid replacement of vegetation with housing and commercial infrastructure reminiscent of what was reported by the respondents may render the cities to be prone to the vagaries of weather/climate-related factors [1]. Atmospheric temperature may increase causing heat islands and heat waves, and torrential rainfall and storms may also result in floods. Niang et al. [34] projected that African cities may be seriously affected by urban heat islands as a result of climate and environmental variability. From the results, what was associated with vegetation, land use/land cover change was the unregulated nature of the built-up areas. This is characteristic of the current rapid urbanization being experienced mainly in the developing world especially in Africa through rural-urban migration [35]. Chin [36] described this sort of urbanization in Africa as “Second Phase,” an assertion supported by Leao et al. [37]. According to Leao et al. [37], this “Second Phase” of urban growth is characterized by limited mobility and lower standard of living of the population.

From the perspectives of the majority of the respondents (79.5%), atmospheric temperature has been rising, whereas rainfall has been on the decline within the same period. Though data was not readily available for analysis to confirm or disagree with the assertions from the respondents regarding rising temperature and dwindling rainfall, many authors had already established these assertions. The World Bank [38], for example, stated that rainfall in southern Ghana was mostly high in the 1960s. This however decreased to very low levels in the late 1970s and in the early 1980s and still continue to decline. Cameron [39] confirmed the findings of World Bank and further stated that temperatures in southern Ghana and all other parts of the country have been rising, while rainfall is declining. Using various scenarios, the World Bank especially discovered that temperature in Accra and Takoradi has been projected to rise by 1.7–2.0°C. These observations have also been made by Environmental Protection Agency [40] and Asante and Amuakwa-Mensah [41]. Rising temperature may lead to increase in health-related problems such as meningitis, as well as higher use of energy by the residents of urban areas [42]. Also, decline in rainfall of the urban areas may lead to increase in dust over the cities leading to the prevalence of respiratory diseases [43].

High mosquito prevalence was observed by the respondents. This according to the respondents has increased from the decades of 1960s to the present. This was corroborated by studies like Fobil et al. [44] and Stoler et al. [45]. Studies have also shown that urban dwellers through their activities have created suitable breeding habitats for mosquitoes [44, 46, 47]. Though rapid urbanization and its associated pollution is supposed to eliminate certain species of mosquito such as* Anopheles* species [48, 49], other species have proliferated profusely in especially in polluted cities of Africa [50]. As the cities expand so are the breeding places for mosquitoes [46]. The fact that respondents could link the poor environmental maintenance to the proliferation of mosquitoes means that they were aware of some important consequences of their actions and this can be taken advantage of in environmental health education. The government of Ghana instituted monthly clean ups in the cities of the country, especially on the first Saturday of every month; however, many residents do not comply with the policy and hence turn-out for the clean-up is always very low. This means that, in spite of the widespread awareness of environmental changes among city dwellers, their commitment to cleaning the environment was low and this needs to be addressed. There is the need for stringent enforcement of laws of sanitation in urban areas of Ghana.

## 5. Conclusion

The study revealed high level of environmental consciousness among the respondents. They were not only aware of the changes in terms of vegetation loss, increases in environmental temperature, and reduction in the rainfall pattern but also clearly understood the factors which contributed to the changes being observed. Interestingly, they could link the changes to the proliferation of mosquitoes. With the high level of consciousness among the communities on environmental changes and the factors contributing to the changes, it is hereby proposed that city authorities should explore the use of community-led efforts in maintaining the environment. This means empowering the communities to take the lead in environmental governance to ensure environmental sustainability.

## Figures and Tables

**Table 1:** Sociodemographic characteristics of the respondents.

Characteristics	Variables	AMA (%)	STMA (%)
Gender	Male	27	26
	Female	23	24

Marital status	Single (never married)	6.1	2.7
	Married	32.7	40.1
	Cohabitation	2	2
	Separated	1.4	2.7
	Divorced	5.4	1.4
	Widowed	3.4	0

Level of education	No education	15.4	12.8
	Primary	16.8	15.4
	Secondary	16.1	17.4
	Tertiary	2	4

Employment	No	36.7	33.3
	Yes	14.3	15.7

Age group	40–44	2	3.3
	45–49	11	7.3
	50–54	12	8
	55–59	8.7	10.7
	60–64	6	6.7
	65–69	4	6
	70+	6	8

Number of years living in the city (year group)	35–39	13.3	10.7
	40–44	8	10
	45–49	10.7	9.3
	50–54	7.3	6
	55–59	4.7	6
	60–64	2	2.7
	65–69	2	1.3
	70+	2	4

	*N* = 150	75	75

**Table 2:** Perceived environmental changes in Accra (AMA) and Sekondi-Takoradi Metropolitan Area (STMA).

Affected environmental features	Proportion of respondents	
	AMA (%)	STMA (%)
Land use/ land cover	93	83
Vegetation	100	96
Streams/rivers	75	81

Ability to remember places which were vegetated in the past	77	86

*N* = 150	75	75

**Table 3:** Indicators of vegetation cover and land use/land cover changes in Accra (AMA) and Sekondi-Takoradi Metropolitan Area (STMA).

Decade	AMA	STMA
	Vegetation/land use/land cover	Vegetation/land use/land cover
1961–1970	(i) Abundant grass/ shrubs (ii) Numerous neem trees *(Azadirachta indica)* (iii) Farming activities (iv) Few houses	(i) Vast forest cover (ii) Numerous coconut trees (iii) Few houses (iv) Farming activities

1971–1980	(i) Increase in housing (ii) Farming activities (iii) Abundant grass/shrubs	(i) Forest depletion begun (ii) Increase in housing (iii) Farming activities

1981–1990	(i) Increase in housing (ii) Rising of slums (iii) Farming activities	(i) Forest depletion increased (ii) Uncoordinated building

1991–2000	(i) Uncoordinated building/rising of shanty towns (ii) A few farming activities	(i) Forest depletion worsen (ii) Extinction of coconut trees

2001–2010	(i) Uncoordinated building (ii) Decrease in vegetation (iii) Increasing commercial activities	(i) Uncoordinated building (ii) Rapid expansion of built-up areas

**Table 4:** Respondents views on environmental weather/climate variability.

Characteristics	Variable	AMA (%)	STMA (%)
Temperature	Temperature has remained constant over the years	8	2.7
	Temperature has fluctuated over the years	20	6.8
	Temperature has consistently been increasing over the years	70.7	89.2
	Temperature has consistently been decreasing over the years	1.3	1.4

Rainfall	Amount of rainfall has remained unchanged over the years	5.5	4.3
	Amount of rainfall has fluctuated over the years	21.9	17.1
	Amount of rainfall has consistently been increasing over the years	5.5	8.6
	Amount of rainfall has consistently been decreasing over the years	67.1	70

	*N* = 150	75	75

**Table 5:** Respondents' views on abundance of mosquitoes in the past decades.

Decade	Extremely abundant (%)	Very abundant (%)	Abundant (%)	Somehow abundant (%)	Not abundant (%)	Do not know (%)	Not applicable (%)
1961–1970	12.3	3.8	5.7	6.6	**30.2**	19.8	21.7
1971–1980	3.8	20.8	7.5	**36.8**	6.6	19	5.5
1981–1990	0.0	10.4	**52.8**	17.9	2.8	16.0	0.0
1991–2000	0.9	**38.7**	19.8	21.7	3.8	15.1	0.0
2001–2010	**34.9**	17.0	6.6	10.4	17.0	14.1	0.0

*N* = 150.

## Data Availability

The datasets analyzed during the current study are available from the corresponding author (PADM) on reasonable request.
